# Changes in (risk) behavior and HPV knowledge among Dutch girls eligible for HPV vaccination: an observational cohort study

**DOI:** 10.1186/s12889-018-5745-6

**Published:** 2018-07-05

**Authors:** Robine Donken, Adriana Tami, Mirjam J. Knol, Karin Lubbers, Marianne A. B. van der Sande, Hans W. Nijman, Toos Daemen, Willibrord C. M. Weijmar Schultz, Hester E. de Melker

**Affiliations:** 1 Center for Infectious Disease Control, National Institute for Public Health and the Environment (RIVM), Bilthoven, the Netherlands; 2 Department of Pathology, VU University Medical Center (VUmc), P.O. Box 7057, 1007 MB Amsterdam, the Netherlands; 3 Department of Medical Microbiology, University of Groningen, University Medical Center Groningen, Groningen, The Netherlands; 4 Julius Center for Health Sciences and Primary Care, University Medical Center Utrecht, Utrecht, the Netherlands; 5 Department of Public Health, Institute of Tropical Medicine, Antwerp, Belgium; 6 Department of Obstetrics and Gynecology, University of Groningen, University Medical Center Groningen, Groningen, The Netherlands

**Keywords:** Human papillomavirus, HPV vaccination, Adolescents, Knowledge, Sexual behavior, Condom use

## Abstract

**Background:**

Implementation of human papillomavirus (HPV) vaccination raised concerns that vaccination could lead to riskier sexual behavior. This study explored how possible differences in sexual behavior and HPV knowledge developed over time between HPV-vaccinated and unvaccinated girls.

**Methods:**

A random sample of 19,939 girls (16–17 year olds) eligible for the catch-up HPV vaccination campaign in the Netherlands was invited for a longitudinal study with questionnaires every 6 months over a two-year follow-up period. Possible differences over time between vaccinated and unvaccinated participants were studied using generalized equations estimation (GEE).

**Results:**

A total of 2989 girls participated in round one, of which 1574 participated (52.7%) in the final 5th round. Vaccinated girls were more likely to live in more urban areas (OR 1.28, 95%CI 1.10–1.47) and to use alcohol (OR 1.46, 95%CI 1.24–1.70) and contraceptives (OR 1.69, 95%CI 1.45–1.97). Vaccinated and unvaccinated girls showed comparable knowledge on HPV, HPV vaccination, and transmission. Vaccinated girls were more likely to be sexually active (OR 1.19, 95%CI 1.02–1.39), and this difference increased over time (OR for interaction 1.06, 95%CI 1.00–1.12). However, they had a slightly lower number of lifetime sexual partners (mean difference − 0.20, 95%CI -0.41-0.00). Vaccinated girls were less likely to use a condom with a steady partner (aOR 0.71, 95%CI 0.56–0.89). However, the difference between vaccinated and unvaccinated girls with regard to condom use with casual or steady partner(s) did not significantly change over time.

**Conclusion:**

Overall, we did not find indications that vaccination influenced sexual behavior in girls during 2 years of follow-up. The few differences found may be related to existing disparities in the socio-demographic characteristics of the young population pointing to the importance and improvement of education with regard to safe sex practices. Our findings do not suggest that vaccination status is associated with changes in sexual risk behavior and thus it is unlikely that this might influence the effectiveness of the vaccination program.

## Background

A persistent infection with a high-risk type of the human papilloma virus (HPV) is the most important risk factor for the development of premalignant cervical intraepithelial neoplasia (CIN) and cervical cancer [1]. HPV is a common sexually transmitted infection (STI), with a lifetime risk of approximately 80% for acquiring an HPV infection in both sexually active males and females [2]. In 2009, girls aged 13 to 16 years in the Netherlands were offered the bivalent HPV vaccine (Cervarix®) during a “catch-up” vaccination program [3]. The vaccine uptake (completely vaccinated with a three-dose schedule) during this “catch-up” campaign was 52.3% [4]. From 2010, HPV vaccination was included in the Dutch National Immunization Program for girls in the year they turn 13 [3]. Implementation of HPV vaccination raised the concern that a vaccine against an STI might lead to more and/or riskier sexual behavior by vaccinated adolescents [5]. Although only 3 to 6% of parents in the United Kingdom stated the above as a reason to refuse vaccination of their daughters [6, 7], 16 to 26% of parents mentioned having this worry [8–12]. Wilde’s Risk Homeostasis Theory suggests that individuals anticipate a lower risk from a certain behavior due to the perceived benefits of that behavior [13]. In the case of HPV vaccination, this could imply that vaccinated individuals might have lower risk perceptions, not only for acquiring HPV, but also other STIs, and therefore show riskier sexual behavior. The development of adequate risk perceptions is related to knowledge regarding HPV and HPV vaccination [14, 15]. Possible differences in behavior or knowledge that exist or may develop over time between vaccinated and unvaccinated girls might influence the outcomes of prevention strategies against HPV-associated diseases [16]. Previous studies exploring sexual behavioral changes by HPV vaccination were generally small or had a short follow-up period [12]. These studies observed either no association for pregnancy and STI rates with HPV vaccination [17–20] or a lower rate of *Chlamydia trachomatis* in the vaccinated group [21]. No association was found for HPV vaccination with ever had sex [5], the number of sexual partners [5, 17, 18, 22, 23] or the consistency of condom use [5, 17, 21, 22]. In this study, we explored to what extent differences between HPV-vaccinated and unvaccinated adolescent girls in sexual behavior and HPV knowledge developed over time (follow-up of 2 years) in the Netherlands.

## Methods

### Study design

Recruitment and methodology of the round one survey among 16 to 17 year old girls has been described previously [24]. Briefly, in December 2010, a random sample of 19,939 girls (both vaccinated and unvaccinated) who had been eligible for the HPV-vaccination catch-up campaign in 2009 was invited by post with an information letter for herself and for her parents/caretakers to participate in a longitudinal online semi-structured questionnaire study [24]. Those consenting to participate were invited another four times (Rounds 2 to 5: July 2011, February and August 2012, and February 2013) to answer similar online questionnaires. Reminders and invitations to participate in these follow-up questionnaires were sent via the online system (EFS Survey version 8.1, Unipark (Questback)). This research was performed in accordance to the principles of the Declaration of Helsinki [25]. Based on the nature of the study, the Dutch Central Committee on Research Involving Human Subjects (Centrale Commissie Mensgebonden Onderzoek (CCMO)) decided that approval from a medical-ethical review committee was not required for this study, in agreement with the Dutch Medical Research involving Human Subjects Act. The CCMO allowed to receive consent through the online system from the participating girls, no written consent from the girls or their parents was required [24].

### Measures

The used questionnaire contained pre-coded questions on socio-demographic characteristics (e.g. educational level, ethnicity, alcohol and smoking behavior), sexual behavior (e.g. ever had sex and number of partners) and questions on HPV infection and vaccination, in order to verify the participants knowledge. Vaccination history of the participants was obtained from the national vaccination database Praeventis [26]. Comparable to Mollers et al.*,* who previously described round one of this study, composite scores for knowledge, assigning a point for each correct answer to questions regarding general knowledge (ten questions), HPV transmission (11 questions), and HPV vaccination (three questions) were calculated [24]. Questions regarding general HPV knowledge, vaccination and transmission were only incorporated in rounds one and five.

### Statistical analysis

We examined whether the trend over time in socio-demographic and sexual risk behavior was different between vaccinated and unvaccinated girls using generalized estimating equation (GEE) models with an exchangeable correlation structure. For dichotomous outcomes, we used a binomial GEE model with logit link, resulting in odds ratios (OR) as the measure of association. For continuous outcomes, a GEE with a normal distribution was used, resulting in a mean difference. For univariable analyses, potential risk factors were used as dependent variables, and vaccination status was used as the independent variable. First, we added time (rounds of interviews, as continuous factor) and the interaction between vaccination status and time as independent variables. Estimates for interaction between vaccination status and time were only reported if a significant interaction (i.e. a difference between vaccinated and unvaccinated changed over time) was observed. If no significant interaction was observed, an overall (across all time-points) estimate for vaccination status was reported. To explore whether a significant development over time in the two groups occurred, we stratified the data for vaccinated and unvaccinated participants for all variables and used time as the independent variable. Similar analyses were performed for the questions regarding knowledge on HPV (vaccination) and transmission, in which the outcome was ‘giving the correct answer’. We adjusted sexual risk behavior and condom use in a multivariable GEE model, correcting for socio-demographic characteristics which were significantly different between vaccinated and unvaccinated participants.. Analyses were performed using SAS 9.3 (SAS Institute, Inc. 2010, USA).

## Results

From 19,939 invited girls, 2989 (15%) participated in round one of this study. [24] Participation in the following rounds was, in chronological order: 2040 (68.3%), 1778 (59.5%), 1789 (59.9%), and 1574 (52.7%) of the 2989 girls who were included in round one.

### Participant characteristics and health behavior

The analysis of socio-demographic characteristics, sexual risk behavior, and HPV knowledge among vaccinated and unvaccinated girls from the first round of study has been previously reported [24]. At the start of the study 65% of all participants were vaccinated, participants had a median age of 17 years, vaccinated participants were more likely to live in more urbanized areas, to ever have used alcohol, contraception and to ever had sex. Table 1 shows the univariable longitudinal analysis (using GEE) of the participant characteristics and health behavior comparing vaccinated and unvaccinated girls. There was a significant difference in the degree of urbanization, alcohol use, and use of contraceptives between vaccinated and unvaccinated girls; these differences did not change over time. Vaccinated girls were more likely to live in more urbanized areas (OR 1.28, 95%CI 1.10–1.47) and to have ever used alcohol (OR 1.46, 95%CI 1.24–1.70) and contraceptives (OR 1.69, 95%CI 1.45–1.97). The percentage of sexually active participants increased significantly over time in both vaccinated and unvaccinated individuals, from respectively 56 to 80% and from 52 to 71% (both *p* < 0.01). Vaccinated girls were more likely than unvaccinated girls to report ever having sex (OR 1.19, 95%CI 1.02–1.39) in round one, and this difference increased over time (significant interaction between vaccination status and time: OR 1.06, 95%CI 1.00–1.12), reaching an OR of 1.49 (95%CI 1.18–1.87) in round five.

**Table 1:** Univariable longitudinal analysis (using GEE) of characteristics of participating girls over time

	Round 1^b^	Round 2	Round 3	Round 4	Round 5		
Category	n (%)	n (%)	n (%)	n (%)	n (%)	*p*-value	OR (+ 95% CI)
Participants							
Unvaccinated	1051 (100)	679 (65)	565 (54)	572 (54)	502 (48)		
Vaccinated	1938 (100)	1361 (70)	1213 (63)	1217 (63)	1072 (55)		
Median age (range)							
Unvaccinated	17 (16–17)	17 (17–18)	18 (18–19)	19 (18–19)	19 (19–20)		
Vaccinated	17 (16–17)	17 (17–18)	18 (18–19)	19 (18–19)	19 (19–20)		
More urban areas (> 1000 inhabitants)							
Unvaccinated	495 (47)	315 (47)	278 (49)	308 (55)	282 (57)	< 0.01	Ref
Vaccinated	1019 (53)	714 (53)	712 (59)	734 (61)	685 (65)	< 0.01	1.28 (1.10–1.47)
Low/Middle Education^a^							
Unvaccinated	434 (42)	235 (35)	189 (34)	180 (32)	153 (31)	0.10	Ref
Vaccinated	779 (40)	465 (35)	394 (33)	386 (32)	324 (30)	0.07	0.96 (0.83–1.11)
Ever have used alcohol							
Unvaccinated	751 (72)	507 (76)	431 (77)	449 (79)	389 (78)	< 0.01	Ref
Vaccinated	1509 (78)	1107 (82)	988 (82)	1021 (84)	888 (83)	< 0.01	1.46 (1.24–1.70)
Ever smoked							
Unvaccinated	261 (25)	176 (26)	152 (27)	55 (10)	52 (10)	< 0.01	Ref
Vaccinated	443 (23)	330 (24)	296 (25)	125 (10)	107 (10)	< 0.01	0.92 (0.79–1.09)
Ever used contraception							
Unvaccinated	640 (64)	443 (68)	395 (71)	420 (75)	359 (75)	< 0.01	Ref
Vaccinated	1380 (72)	1038 (78)	973 (81)	1020 (85)	889 (86)	< 0.01	1.69 (1.45–1.97)
Ever had sex							
Unvaccinated	525 (52)	390 (60)	362 (65)	383 (70)	349 (71)	< 0.01	Ref
Vaccinated	1070 (56)	845 (64)	845 (71)	914 (76)	833 (80)	< 0.01	(^c^1) 1.19 (1.02–1.39)
							(^c^2) 1.30 (1.24–1.35)
							(^c^3) 1.06 (1.00–1.12)

The *p*-value indicates whether there is a significant change over time. The OR indicates possible differences between vaccinated and unvaccinated participants overall (across all five time points)

^a^Low/Middle = no/primary education, lower general to intermediate vocational secondary education; High = higher vocational/general secondary education, (pre)university education

^b^Previously published by Mollers et al. [17]

^c^Significant interaction time and vaccination status; 1: OR for vaccination status at round one, 2: OR for round, 3: OR for interaction between vaccination status and round

### Sexual risk factors among sexually active participants

As none of the interaction terms between time and vaccination status were significant for the behaviors reported in Table 2, there was no evidence for a difference between vaccinated and unvaccinated sexually active participants in changed sexual behavior. However, among sexually active participants in both groups, sexual behavior changed over time compared to round one, as the lifetime number of partners increased and the percentage of participants with a casual partner declined. The lifetime number of sexual partners was slightly lower among vaccinated girls (adjusted mean difference − 0.26, 95%CI -0.46-0.05) (Table 2).

**Table 2:** Univariable longitudinal analysis (using GEE) of sexual risk behavior factors among sexually active participants

	Round 1^a^	Round 2	Round 3	Round 4	Round 5			
Category	n (%)	n (%)	n (%)	n (%)	n (%)	*p*-value	OR (+ 95% CI)	aOR^c^ (+ 95% CI)
Having had sex for the first time during previous 6 months								
Unvaccinated	–	84 (22)	71 (20)	69 (18)	59 (17)	0.01	Ref	Ref
Vaccinated	–	216 (26)	166 (20)	170 (19)	179 (21)	< 0.01	1.15 (0.97–1.37)	1.14 (0.96–1.35)
Lifetime number of sexual partners^b^ (mean 95%CI)								
Unvaccinated	2.2 (2.0–2.4)	2.5 (2.3–2.7)	2.7 (2.4–3.0)	3.0 (2.7–3.3)	3.4 (3.1–3.8)	< 0.01	Ref	Ref
Vaccinated	1.9 (1.8–2.0)	2.1 (2.0–2.3)	2.4 (2.3–2.6)	2.9 (2.7–3.1)	3.0 (2.8–3.2)	< 0.01	− 0.20 (− 0.41–0.00)^b^	−0.26 (− 0.46 – -0.05)
Having a steady partner at the moment								
Unvaccinated	349 (67)	275 (71)	247 (68)	275 (72)	250 (72)	0.17	Ref	Ref
Vaccinated	729 (68)	586 (69)	576 (68)	622 (68)	578 (69)	0.34	0.96 (0.82–1.13)	1.02 (0.87–1.19)
Having had a casual partner during previous 6 months								
Unvaccinated	271 (52)	96 (25)	77 (21)	82 (22)	81 (23)	< 0.01	Ref	Ref
Vaccinated	512 (48)	163 (19)	170 (20)	234 (26)	189 (23)	< 0.01	0.92 (0.80–1.06)	1.11 (0.96–1.29)

The *p*-value indicates whether there is a significant change over time. The OR indicates possible differences between vaccinated and unvaccinated participants overall (across all five time points)

- Not asked in this round (Having had sex for the first time during previous 6 months was only questioned from round two onward)

^a^Previously published by Mollers et al. [17]

^b^For continuous variables, the mean difference was calculated. For other variables, odds ratios were calculated

^c^OR was adjusted for degree of urbanization and alcohol use

No significant interactions between time and vaccination status were observed for condom use with casual and/or steady partners. Condom use with a casual partner did not change significantly over time in vaccinated or unvaccinated participants. There was no difference in always using a condom with a casual partner in the preceding 6 months between vaccinated and unvaccinated participants (adjusted OR 1.00, 95%CI 0.78–1.27). However, condom use with a steady partner declined over time in both vaccinated and unvaccinated participants. In addition, vaccinated girls were less likely to always use a condom with a steady partner (adjusted OR 0.71, 95%CI 0.57–0.90). Although a difference in change over time was observed between vaccinated and unvaccinated when examining the variable ‘always using condoms’ (OR for interaction 0.91; 95%CI 0.82–1.00), after adjustment for other variables no significant change in difference (interaction) was observed anymore (OR for vaccination 0.85; 95%CI 0.70–1.02) (Table 3).

**Table 3:** Condom use with steady or casual partner among sexually active participants

	Round 1^a^	Round 2	Round 3	Round 4	Round 5		OR (+ 95% CI)	aOR^b^ (+ 95%CI)
Category	n (%)	n (%)	n (%)	n (%)	n (%)	*p*-value		
Always using a condom with steady partner during previous 6 months								
Unvaccinated	67 (19)	59 (22)	41 (17)	40 (14)	34 (14)	< 0.01	Ref	Ref
Vaccinated	126 (17)	77 (13)	79 (14)	65 (10)	53 (9)	< 0.01	0.71 (0.57–0.89)	0.71 (0.57–0.90)
Always using a condom with a casual partner during previous 6 months								
Unvaccinated	75 (28)	27 (28)	17 (22)	27 (32)	20 (26)	0.12	Ref	Ref
Vaccinated	144 (28)	50 (31)	51 (30)	67 (29)	44 (23)	0.97	1.00 (0.79–1.28)	1.00 (0.78–1.28)
Always using a condom during previous 6 months (combined for casual and steady partner)								
Unvaccinated	93 (20)	75 (22)	56 (18)	59 (18)	50 (16)	< 0.01	Ref	Ref
Vaccinated	197 (21)	113 (17)	117 (16)	107 (14)	90 (12)	0.02	(^c^1) 1.01 (0.80–1.27)	0.85 (0.70–1.02)
							(^c^2) 0.91 (0.85–0.99)	
							(^c^3) 0.91 (0.82–1.00)	

The *p*-value indicates whether there is a significant change over time. The OR indicates possible differences between vaccinated and unvaccinated participants overall (across all time points)

^a^Previously published by Mollers et al. [17]

^b^OR adjusted for urbanisation degree and alcohol use

^c^Significant interaction between time and vaccination status; 1: OR for vaccination status at round one, 2: OR for round 3, 3: OR for interaction between vaccination status and round

### HPV general knowledge

There were no significant differences in general HPV knowledge score (giving the correct answer) between vaccinated and unvaccinated participants (mean difference 0.11, 95%CI -0.02-0.23) (Table 4). In both groups, the general knowledge score increased significantly (*p* < 0.01); however, no differences in change over time were observed between vaccinated and unvaccinated girls (no significant interaction).

**Table 4:** General knowledge (percentage with correct answer) on HPV and cervical cancer among vaccinated and unvaccinated participants on the first and last rounds

	Round 1^a^	Round 5		
Category	n (%)	n (%)	*p*-value	Mean difference (+ 95% CI)
General knowledge score (mean 95%CI)				
Unvaccinated	4.25 (4.14–4.36)	4.70 (4.54–4.87)	< 0.01	Ref
Vaccinated	4.29 (4.21–4.37)	4.92 (4.80–5.03)	< 0.01	0.11 (− 0.02–0.23)
HPV infections are easily treatable (No)				
Unvaccinated	142 (14)	96 (20)		
Vaccinated	284 (15)	279 (27)		
HPV infections are rare (No)				
Unvaccinated	428 (42)	232 (48)		
Vaccinated	882 (46)	554 (53)		
An HPV infection always leads to cervical cancer (No)				
Unvaccinated	690 (68)	389 (80)		
Vaccinated	1294 (64)	844 (81)		
Cervical cancer is always fatal (No)				
Unvaccinated	835 (82)	427 (88)		
Vaccinated	1579 (82)	923 (89)		
Cervical cancer is easily treatable (No)				
Unvaccinated	178 (18)	108 (22)		
Vaccinated	408 (21)	273 (26)		
Cervical cancer is a common disease (No)				
Unvaccinated	237 (23)	116 (24)		
Vaccinated	334 (17)	238 (23)		
If you have unprotected sex, you are at high risk of an HPV infection (Yes)				
Unvaccinated	732 (72)	345 (71)		
Vaccinated	1411 (74)	782 (75)		
An HPV infection is a risk for cervical cancer (Yes)				
Unvaccinated	815 (80)	400 (82)		
Vaccinated	1555 (81)	888 (85)		
An HPV infection can cause genital warts (Yes)				
Unvaccinated	193 (19)	134 (28)		
Vaccinated	389 (20)	274 (26)		
An HPV infection usually disappears on its own (Yes)				
Unvaccinated	56 (6)	42 (9)		
Vaccinated	80 (4)	61 (6)		

Questions regarding general knowledge were only incorporated in the questionnaires of round one (first) and round five (last). The *p*-values of vaccinated and unvaccinated girls indicate whether the knowledge changed over time within these groups

^a^Previously published by Mollers et al. [17]

### HPV transmission knowledge

Knowledge of HPV transmission (transmission knowledge score) increased significantly over time among vaccinated girls (*p* = 0.01), but not among unvaccinated girls (*p* = 0.60). However, we did not observe a difference in transmission knowledge over time between vaccinated and unvaccinated girls (no significant interaction) (Tables 5 and 6).

**Table 5:** Transmission knowledge (percentage of correct answers) among both vaccinated and unvaccinated participants in the first and last rounds

	Round 1^a^	Round 5		
Category	n (%)	n (%)	*p*-value	Mean difference (+ 95% CI)
Transmission knowledge score (mean 95%CI)				
Unvaccinated	7.47 (7.39–7.55)	7.52 (7.40–7.63)	0.60	Ref
Vaccinated	7.39 (7.33–7.45)	7.53 (7.46–7.61)	0.01	0.01 (−0.10–0.10)
HPV can be transmitted by				
Holding Hands (No)				
Unvaccinated	1002 (99)	481 (99)		
Vaccinated	1882 (99)	1034 (99)		
Deep throat kissing (No)				
Unvaccinated	911 (90)	449 (92)		
Vaccinated	1711 (90)	968 (93)		
Skin-to-skin contact (Yes)				
Unvaccinated	95 (9)	36 (7)		
Vaccinated	185 (10)	71 (7)		
Stroking partner at genitals (Yes)				
Unvaccinated	340 (34)	126 (26)		
Vaccinated	587 (31)	258 (25)		
Public toilet (No)				
Unvaccinated	836 (83)	427 (88)		
Vaccinated	1575 (82)	927 (89)		
Unprotected oral sex (Yes)				
Unvaccinated	631 (62)	294 (61)		
Vaccinated	1219 (64)	640 (62)		
Unprotected vaginal sex (Yes)				
Unvaccinated	967 (95)	469 (96)		
Vaccinated	1818 (95)	1004 (97)		
Unprotected anal sex (Yes)				
Unvaccinated	718 (71)	349 (70)		
Vaccinated	1345 (70)	766 (71)		
Sex with a condom (Yes)				
Unvaccinated	156 (15)	95 (20)		
Vaccinated	238 (12)	157 (15)		
Sharing a spoon or cup (No)				
Unvaccinated	962 (95)	464 (95)		
Vaccinated	1777 (93)	999 (96)		
Sneezing/coughing (No)				
Unvaccinated	959 (95)	470 (97)		
Vaccinated	1794 (93)	1010 (97)		

Questions regarding transmission knowledge were only incorporated in the questionnaires of round one (first) and round five (last). The *p*-values of vaccinated and unvaccinated indicate whether the knowledge changed over time within these groups

^a^Previously published by Mollers et al. [17]

**Table 6:** Knowledge of the consequences (percentage of correct answers) of HPV vaccination reported by vaccinated and unvaccinated girls

	Round 1^a^	Round 2	Round 3	Round 4	Round 5		
Category	n (%)	n (%)	n (%)	n (%)	n (%)	*p*-value	Mean difference (+ 95% CI)
HPV vaccination score (mean 95%CI)							
Unvaccinated	2.50 (2.45–2.54)	2.46 (2.40–2.52)	2.59 (2.53–2.65)	2.60 (2.54–2.65)	2.67 (2.62–2.73)	< 0.01	Ref
Vaccinated	2.44 (2.41–2.47)	2.40 (2.36–2.43)	2.50 (2.46–2.53)	2.54 (2.51–2.57)	2.67 (2.64–2.71)	< 0.01	(^b^1) -0.07 (−0.12--0.01)
							(^b^2) 0.03 (0.02–0.04)
							(^b^3) 0.02 (0.00–0.04)
Vaccination protects against all HPV types (No)							
Unvaccinated	701 (70)	416 (63)	384 (69)	388 (69)	360 (75)		
Vaccinated	1110 (58)	638 (48)	665 (55)	697 (58)	753 (73)		
HPV vaccination protects against all STIs (No)							
Unvaccinated	865 (86)	574 (88)	518 (93)	520 (93)	454 (95)		
Vaccinated	1702 (89)	1246 (94)	1144 (95)	1159 (97)	1008 (97)		
Condoms are not needed anymore once vaccinated (No)							
Unvaccinated	939 (94)	623 (95)	542 (97)	545 (98)	468 (98)		
Vaccinated	1842 (97)	1297 (98)	1187 (99)	1186 (99)	1016 (98)		

The *p*-values of vaccinated and unvaccinated indicate whether the knowledge changed over time within these groups

^a^Previously published by Mollers et al. [17]

^b^Significant interaction between time and vaccination status; 1: difference for vaccination status at round one, 2: difference for round, 3: difference for interaction between vaccination status and round

### Knowledge of the consequences of HPV vaccination

In round one, vaccinated participants had a lower overall knowledge score regarding the effects of HPV vaccination (mean difference − 0.07, 95%CI -0.12 - -0.01) (Table 6). However, over time, this difference diminished (interaction term between time and vaccination 0.02, 95%CI 0.00–0.04). In round five, there was no significant difference between vaccinated and unvaccinated girls (mean difference 0.01, 95%CI -0.04-0.07). In both vaccinated and unvaccinated participants, the knowledge score regarding HPV vaccination significantly increased over time.

## Discussion

Our main hypothesis was that vaccinated girls might perceive themselves at a lower risk for contracting STIs and therefore develop higher risk sexual behaviors, for instance, by lowering condom use. Although we observed that vaccinated girls were less likely to use a condom with their steady partner, changes in condom use over time with both steady and casual partners did not differ between vaccinated and unvaccinated girls. Vaccinated girls were more likely to ever have been sexually active, and this difference increased over time, but among sexually active participants, we did not observe noteworthy differences in sexual behavior over time between vaccinated and unvaccinated girls. Also, although knowledge of our participants on HPV, HPV transmission and vaccination was suboptimal, we did not find major differences between vaccinated and unvaccinated girls.

We did not find strong indications for a difference in change in condom use between vaccinated and unvaccinated over time as there were no differences in condom use overall or with a casual partner. However, vaccinated women were less likely to report to always use a condom with their steady partner. In general, the decline in condom use for both vaccinated and unvaccinated girls over time might be explained by longer duration of relationships and/or a switch to other contraceptives. Indeed, use of contraceptives other than condoms, increased significantly in both vaccinated and unvaccinated participants over time (data not shown). Educating the adolescent population on sexual risk remains of critical importance. Previous studies on condom use and a possible difference between vaccinated and unvaccinated women showed either no difference in condom use [18, 23, 27–29] or a higher condom use among vaccinated women [17, 22, 30, 31]. A recent systematic review incorporating 21 studies did not find evidence that sexual risk compensation or disinhibition was associated with HPV vaccination [32]. Using data from insurance databases, Jena et al. also observed that there was no association between HPV vaccination and an increase in STI rates among 12 to 18 year-old females in the United States [33].

Vaccinated girls were more likely to be sexually active, and this also increased more over time than among unvaccinated girls in our study. The proportion of sexually active vaccinated women in this age group was comparable to what was observed in a representative sample of the Netherlands in 2005 and 2012 (18–20 years: 76 and 77%) [34, 35]. Also, when the model for ever had sex was adjusted for other characteristics showing a difference between vaccinated and unvaccinated participants (urbanization degree, level of education and contraceptive use), the adjusted OR was 1.03 (95%CI 0.89–1.19), indicating that there was no difference in the proportion of participants ever had sex between the vaccinated and unvaccinated participants. We found that vaccinated girls who were sexually active reported a lower number of lifetime sexual partners, and this difference did not change over time. Previously, several studies examined the relationship between the number of partners and HPV vaccination. Most of these studies did not find an association between the number of partners and vaccination status or observed a lower number of partners among vaccinated women, which is in line with our results [18, 21–23, 28–31, 36, 37].

We observed some differences between vaccinated and unvaccinated participants in the degree of urbanization, alcohol and contraceptive use, being sexually active, and vaccination knowledge, but these differences did not change over time. Given the vaccination uptake of approximately 50% for the first dose in these birth cohorts [38], vaccinated and unvaccinated girls may differ by nature prior to vaccination, and changes over time in behavior among either group might reflect an impact of vaccination or the influence of underlying differences between these groups. Differences in these socio-demographics between vaccinated and unvaccinated girls might lead to differences in sexual behavior.

In general, we did not observe differences (over time) in general HPV or transmission knowledge score between vaccinated and unvaccinated individuals. However, HPV knowledge in both vaccinated and unvaccinated girls could be improved. These findings were mainly in line with the studies of Lenselink et al. and Sopracordevole et al. [39, 40]. We observed a small difference in the HPV knowledge vaccination score between vaccinated and unvaccinated participants; however, this difference diminished during follow-up. In this respect, vaccinated girls were less likely to know that HPV vaccination does not protect against all HPV types (data not shown). It will be worthwhile to focus specifically on this topic in future communications, as it might influence participation in cervical cancer screening at a later age because vaccinated women might think they are no longer at risk.

Our study had some weaknesses and several strengths. Unfortunately, the response rate was only 15%, and the overall drop-out rate was 45%. Also, like as many other studies questioning behavior, recall bias on sexual behavior could have occurred in this study, although this is unlikely to be different for vaccinated and unvaccinated participants. While previous studies did not find evidence for behavioral risk disinhibition following vaccination, this might have been due to either the cross-sectional design or the limited power. Our large prospective study has now provided a much more robust basis for the lack of association. The vaccination status of participants was obtained from the Dutch vaccination registry and not dependent on self-reporting. Another strength is that we combined assessment of sexual behavior with assessment of participants’ knowledge on HPV and transmission of HPV.

## Conclusion

During our two-years of follow-up e found that vaccinated girls were less likely to use a condom with their steady partner, but comparable condom use was observed for casual partners between vaccinated and unvaccinated girls. The few observed differences between the groups and the low knowledge in both groups on HPV underline the importance for more attention to safe sex practices. Our findings together with those from other previous studies do not imply that vaccination status is related to changes in sexual risk behavior hence, it is unlikely that this might influence the effectiveness of the vaccination program.

## Abbreviations

aOR: Adjusted odds ratio
CI: Confidence interval
CIN: Cervical intraepithelial neoplasia
GEE: Generalized estimations equations
HPV: Human papillomavirus
OR: Odds ratio
STI: Sexually transmitted infections
